# *Pseudomonas aeruginosa* L10: A Hydrocarbon-Degrading, Biosurfactant-Producing, and Plant-Growth-Promoting Endophytic Bacterium Isolated From a Reed (*Phragmites australis*)

**DOI:** 10.3389/fmicb.2018.01087

**Published:** 2018-05-25

**Authors:** Tao Wu, Jie Xu, Wenjun Xie, Zhigang Yao, Hongjun Yang, Chunlong Sun, Xiaobin Li

**Affiliations:** ^1^Shandong Provincial Engineering and Technology Research Center for Wild Plant Resources Development and Application of Yellow River Delta, College of Biological and Environmental Engineering, Binzhou University, Binzhou, China; ^2^Shandong Key Laboratory of Eco-Environmental Science for the Yellow River Delta, Binzhou University, Binzhou, China; ^3^Department of Bioengineering, Binzhou Vocational College, Binzhou, China; ^4^State Key Laboratory of Microbial Metabolism, School of Life Sciences and Biotechnology, Shanghai Jiao Tong University, Shanghai, China

**Keywords:** endophytic bacterium, *Pseudomonas aeruginosa*, complete genome, hydrocarbon degradation, biosurfactant synthesis, plant growth promotion

## Abstract

Bacterial endophytes with the capacity to degrade petroleum hydrocarbons and promote plant growth may facilitate phytoremediation for the removal of petroleum hydrocarbons from contaminated soils. A hydrocarbon-degrading, biosurfactant-producing, and plant-growth-promoting endophytic bacterium, *Pseudomonas aeruginosa* L10, was isolated from the roots of a reed, *Phragmites australis*, in the Yellow River Delta, Shandong, China. *P. aeruginosa* L10 efficiently degraded C_10_–C_26_ *n*-alkanes from diesel oil, as well as common polycyclic aromatic hydrocarbons (PAHs) such as naphthalene, phenanthrene, and pyrene. In addition, *P. aeruginosa* L10 could produce biosurfactant, which was confirmed by the oil spreading method, and surface tension determination of inocula. Moreover, *P. aeruginosa* L10 had plant growth-stimulating attributes, including siderophore and indole-3-acetic acid (IAA) release, along with 1-aminocyclopropane-1-carboxylic (ACC) deaminase activity. To explore the mechanisms underlying the phenotypic traits of endophytic *P. aeruginosa* L10, we sequenced its complete genome. From the genome, we identified genes related to petroleum hydrocarbon degradation, such as putative genes encoding monooxygenase, dioxygenase, alcohol dehydrogenase, and aldehyde dehydrogenase. Genome annotation revealed that *P. aeruginosa* L10 contained a gene cluster involved in the biosynthesis of rhamnolipids, *rhlABRI*, which should be responsible for the observed biosurfactant activity. We also identified two clusters of genes involved in the biosynthesis of siderophore (*pvcABCD* and *pchABCDREFG*). The genome also harbored tryptophan biosynthetic genes (*trpAB, trpDC, trpE, trpF*, and *trpG*) that are responsible for IAA synthesis. Moreover, the genome contained the ACC deaminase gene essential for ACC deaminase activity. This study will facilitate applications of endophytic *P. aeruginosa* L10 to phytoremediation by advancing the understanding of hydrocarbon degradation, biosurfactant synthesis, and mutualistic interactions between endophytes and host plants.

## Introduction

Soil contaminated by petroleum hydrocarbons has posed a serious threat to human health and ecological security, and represents a serious environmental problem (Bortey-Sam et al., 2014; Shi et al., 2015). Compared with the chemical and physical remediation technologies, bioremediation strategies are regarded as the cost-effective and sustainable alternatives for soil remediation (Chen et al., 2015). Among bioremediation strategies, phytoremediation, or utilizing growing plants to reduce the concentrations of organic and inorganic pollutants in soil, is a promising technology (Truu et al., 2015). However, the presence of organic pollutants including petroleum hydrocarbons in soil actually decreases plant growth (Gerhardt et al., 2009), and survival of plants becomes tough and then its overall health under pollutant stress is impaired (Mishra et al., 2017). The combined use of plants and bacteria, based on the synergistic activities of plants and their associated microbes, has been recently proposed to enhance the efficiency of remediation of soil contaminated with organic pollutants (Berg, 2009; Arslan et al., 2017). In the plant-microbe-based remediation system, plants provide residency and nutrients to their associated endophytic and rhizosphere bacteria (Arslan et al., 2017). In return, the bacteria support plant growth by the degradation and detoxification of hydrocarbons (Tara et al., 2014). Moreover, they improve plant growth and health due to their innate plant growth-promoting mechanisms (Pawlik et al., 2017).

Recently, plant–endophyte partnership has gained popularity compared to other mechanisms of bioremediation (Zhu et al., 2014). Host plants may take up hydrocarbons from the soils and translocate in their different tissues in which endophytic bacteria reside (Chhikara et al., 2010). A major advantage of endophytic bacteria over rhizobacteria or soil bacteria is that they reside in internal tissues of the host plant and hence have less competition for nutrients and space (Doty, 2008). Endophytic bacteria have a greater capacity to enhance petroleum hydrocarbon phytoremediation than rhizosphere or soil bacteria (Barac et al., 2004; Doty, 2008; Weyens et al., 2010; Balseiro-Romero et al., 2016). However, insufficient knowledge of the mechanism underlying the interactions between plants and hydrocarbon-degrading and/or plant-growth-promoting endophytic bacteria (Feng et al., 2017), along with a lack of genetic data, has hampered the application of endophytes in phytoremediation. The application of high-throughput sequencing techniques and functional genomics has been providing a new avenue for a deeper understanding of the interactions between microbes and their plant hosts.

Therefore, in the present study, a hydrocarbon-degrading, biosurfactant-producing, plant-growth-promoting, and endophytic *Pseudomonas aeruginosa* L10 was isolated from the roots of a reed (*Phragmites australis*) that grows in high-salinity oil-polluted areas of the Yellow River Delta, Shandong, China. To the best of our knowledge, there is no previous report of an endophytic *P. aeruginosa* isolated from a halophytic reed with the characteristics of hydrocarbon degradation, biosurfactant production, and plant growth promotion. In order to gain a deeper understanding of the genetic mechanisms of degrading petroleum hydrocarbons, producing biosurfactants, and promoting plant growth, we sequenced the complete genome of the endophytic *P. aeruginosa* L10. We identified genes related to petroleum hydrocarbon degradation, biosurfactant synthesis, and plant growth promotion.

## Materials and Methods

### Sampling and Isolation of Endophytic Bacteria

Healthy plant samples of reeds (*Phragmites australis*) were collected from region of Shengli Oilfield (the second largest oil-producing base of China), located in the Yellow River Delta, China (N 37°47′05.9″, E 118°39′28.3). This area is characterized by saline-alkaline soils (Gao et al., 2015) and the collected halobiotic reeds grow in salinity areas polluted by oil. Each reed sample was immediately placed in a sterile polyethylene bag, then transported to the laboratory in an ice cooler. Plant samples were treated immediately when they were transported to laboratory.

Reeds were washed with running water for 30 min, and then rinsed with distilled water three times (3 min per wash). After absorbing moisture from the plant surface, the plants were sectioned into roots, stems, and leaves. The plant parts were first surface-sterilized by soaking each plant component in 70% ethanol solution for 2 min, then washed with sterile water three times. Moreover, each plant part was immersed and shaken in 1.2% sodium hypochlorite solution (NaClO) for 30 min, and then washed with sterile water three times (2 min per wash). The last sterile water rinse (100 μL) was plated onto Luria-Bertani (LB) agar medium and incubated for 24 h (at 30°C) in order to test the efficacy of disinfection for each plant part surface (Dai et al., 2016).

Ten volumes of phosphate buffer were added to the sterilized plant tissue. After grinding the tissue completely and letting it stand for 5 min, the suspension was placed on mineral salts (MS) (Mueller et al., 1989) agar plates, supplemented with diesel oil (5 g/L) as the sole source of carbon and energy, and incubated at 30°C for 4 days. Diesel oil was chosen as carbon source because it is a mixture of different hydrocarbon pollutants that have been frequently reported as environmental contaminants (Gallego et al., 2001). The bacterial colonies were selected, sub-cultured, purified, and used for further studies.

### Screening for Effective Hydrocarbon-Degrading and Plant-Growth-Promoting Endophytic Bacteria

Effective hydrocarbon-degrading bacteria were selected for their capacity to degrade diesel oil in liquid medium. Plant-growth-promoting bacteria were selected for their capacity to produce 1-aminocyclopropane-1-carboxylate (ACC) deaminase, siderophore, as well as indole-3-acetic acid (IAA).

### Evaluation of Diesel Oil-Degrading Ability

Ability of isolates to degrade diesel oil was investigated according to a modified method described by Zhang et al. (2010). Diesel oil was sterilized using a 0.22 μm organic filter and added to MS liquid medium to a final concentration of 5 g/L. Bacterial suspensions (MS liquid medium, ∼1 × 10^8^ CFU/mL) of a single strain were added at a 5% inoculum size to the medium, and incubated for 7 days at 30°C, with shaking at 200 revolutions/min (rpm). After incubation, the culture broth was extracted twice with *n*-hexane. All extracts were dehydrated with anhydrous sodium sulfate and evaporated to a final volume of 2 mL with a gentle nitrogen stream in a fume hood. The total petroleum hydrocarbons (TPHs) were quantified by a gas chromatographer with flame ionization detection (GC-FID, Agilent 7890A, United States). A control experiment was performed by inoculating with the boiled cells of isolate. The parameters for the chromatographer were as follows: an Agilent HP-5 GC column was used, with the dimensions 30 m × 0.25 mm × 0.25 μm; helium was used as the carrier gas; the inlet temperature and detector temperature were 290°C and 300°C, respectively; the column oven temperature was maintained at 50°C for 2 min, then increased to 300°C at a rate of 6°C per min, and held at 300°C for 16 min. The total area of detected hydrocarbon peaks was defined as the concentration of TPHs. The degradation ratio of TPHs of diesel oil was calculated according to the formula (1):

(1) $$\text{D}(\%)\;\text{=}\;({\text{C}}_{\text{0}}\;{\text{-C}}_{\text{i}}){\text{/C}}_{\text{0}}\;\times \;\text{100}$$

where D refers to the degradation rate of diesel in the medium; C_0_ refers to the content of residual diesel in the medium inoculated with the boiled cells of isolate; and C_i_ refers to the content of residual diesel in the medium inoculated with the strain. Three replicates were performed for these experiments, and the results are reported as means and standard deviations.

For identification of *n*-alkane peaks in the diesel oil gas chromatogram, a standard mixture of *n*-alkanes (n-C_8_ to n-C_40_) was used as the external standards.

### Measurement of ACC Deaminase Activity

The ACC deaminase activity of bacterial endophytes was screened based on the ability to use ACC as a sole nitrogen source. The ACC deaminase activity was screened according to Penrose and Glick (2003) using the nitrogen-free DF salts minimal agar medium. The solid medium was supplemented with either 2 g/L (NH_4_)_2_SO_4_ or 3 mM ACC as sole nitrogen source, and incubated aerobically. ACC deaminase activity of cell-free extracts was quantitatively determined by measuring the production of alpha-ketobutyrate (alpha-KB) that is generated by the cleavage of ACC by ACC deaminase. After determining the amount of protein and alpha-KB, the enzyme activity was expressed as micromoles of alpha-KB per mg of protein per hour.

### Assay for Siderophore Production

Bacterial endophytes were assayed for siderophores production on chrome azurol S (CAS) agar plate described by Machuca and Milagres (2003). Chrome azurol S agar plates were prepared and divided into equal sectors, spot inoculated with test organism (10 mL of 10^8^ CFU/mL), and incubated at 30°C for 48 h. Development of yellow-orange halo around the growth was considered as positive for siderophore production. By measuring and comparing the diameters of yellow–orange halos, endophytes with the function of siderophore production were screened.

### Assay of IAA Production

The IAA production of the bacterial endophyte was determined according to Sarwar and Kremer (1995) using a microplate method. Briefly, 150 μL of culture filtrate were dispensed into wells of 96-well microplates followed by addition of 100 μL of Salkowski reagent, allowed to react 30 min, and color intensity measured at 530 nm on a microplate reader. The amount of IAA produced was quantified by comparing with the standard curve prepared with known concentrations of IAA. Each experiment consisted of microplates prepared with newly dispensed reagents and filtrates. Comparative experiments were then repeated twice.

### Detection of Biosurfactant Production

Biosurfactant production was detected by the oil spreading method (Anandaraj and Thivakaran, 2010) and surface tension determination of inocula (Zhu et al., 2014). The screened strains above were cultured in MS liquid medium (glucose as carbon source and urea as nitrogen source) on rotary shaker (200 rpm) at 30°C for 2 days. The supernatant was used to determine the diameter of oil spreading and its surface tension. Mineral salt medium without strains, under the same procedure, was used as the control sample (CK). Surface tension was measured in the cell free supernatant using a K100 tensiometer (Kruss GmbH, Hamburg, Germany), following the du Noüy ring method (Górna et al., 2011). Each experiment was repeated thrice, and interpretations were made using the averages of the triplicate measurements.

### Assay for Polycyclic Aromatic Hydrocarbon (PAH) Biodegradation

The ability of endophytic isolates to degrade PAHs was also tested. Cell suspensions (MS liquid medium, ∼ 1 × 10^8^ CFU/mL) of tested bacteria were added at a 5% inoculum volume in 100 mL MS (pH 7.4) supplemented with naphthalene, phenanthrene, or pyrene, respectively. The final concentration of PAH was 200 mg/L. The cultures were incubated at 30°C in a shaker at 200 rpm and extracted after 10 days of incubation. All samples were extracted and analyzed using gas chromatography, according to the methods described above (Zhang et al., 2010).

### Pot Trial Experiment for Assessment of Plant Growth Promotion of Endophytes

The pot trial experiment had two treatments (with and without endophytic inoculation) each with six replicates having seven plantlets in each pot and arranged in a randomized manner. The soil used in the pot trial experiment was treated by the addition of crude oil with the final TPH concentration as 10,527 mg/kg. Overnight bacterial cultures were harvested by centrifugation (6000 × *g*, 5 min). Pellets were washed three times, and suspended in 1/2 strength Hoagland’s solution (Sionit et al., 1981) with cell density as 10^5^–10^6^ CFU/mL. For the colonization of tested endophytes in the plantlets, the plantlets were cultured in 1/2 strength Hoagland’s solution for 7 days. The uniform sized (plant height 10–13 cm) reed plantlets (*Phragmites australis*), both inoculated and uninoculated treatments, were transferred into pottery pots (diameter of 38 cm and depth of 29 cm). Each pottery pot contained 15 kg of sterilized soil. The plantlets were watered every other day. The plants were harvested after 120 days and biomasses (dry weights) of plants from different treatments were measured. Meanwhile, petroleum residues in soil samples were extracted with methylene chloride and the amount of residual TPH was determined gravimetrically (Wu et al., 2012).

### Genome Sequencing, Assembly, and Annotation

In order to more pertinently understand the genetic mechanisms of endophytes to degrade petroleum hydrocarbons, produce biosurfactants, and promote plant growth, we selected one of the most promising endophytic strains. Subsequently, complete genome sequencing of the endophytic bacterium was performed. The genomic DNA of this endophytic strain was isolated from the overnight culture using the method described by Pitcher et al. (1989). The genome of the strain was sequenced using an Illumina HiSeq 4000 sequencing platform, and a PacBio RSII sequencing platform. Sequencing was performed at BGI-Shenzhen, Shenzhen, China. HiSeq raw and PacBio raw data were used to *de novo* assemble the bacterial genome. Gene prediction and annotation were performed on the genome by both the BGI-Shenzhen system and the NCBI Prokaryotic Genome Annotation Pipeline. Predicted proteins were assigned Clusters of Orthologous Groups numbers (Tatusov et al., 2000) and Protein Families domains by querying their sequences against the COG Database and the Pfam database, respectively.

### Genomic Comparative Analysis

We retrieved 100 completely sequenced *P. aeruginosa* genomes from NCBI Genome (last accessed in March 2017) and compared them to the L10 genome sequenced in the present study. Single nucleotide polymorphism (SNP) calling and phylogenetic analysis was performed using kSNP3.0 (Gardner et al., 2015). The kSNP-generated maximum-likelihood tree was visualized using iTOL v3 (Letunic and Bork, 2016). A dot plot comparison of the genomes of *P. aeruginosa* L10 and the reference strain, PAO1, was performed. Nucleotide-based alignments were performed with MUMmer version 3.23 (Kurtz et al., 2004). The dot plots were generated with ‘mummerplot’ and ‘gnuplot’ from the MUMmer package, version 3.23.

### Nucleotide Sequence Accession Number

The genome sequence of *P. aeruginosa* L10 has been submitted to GenBank under accession number CP019338.

## Results

### Isolation and Characterization of Endophytic Isolate L10

**FIGURE 1:**
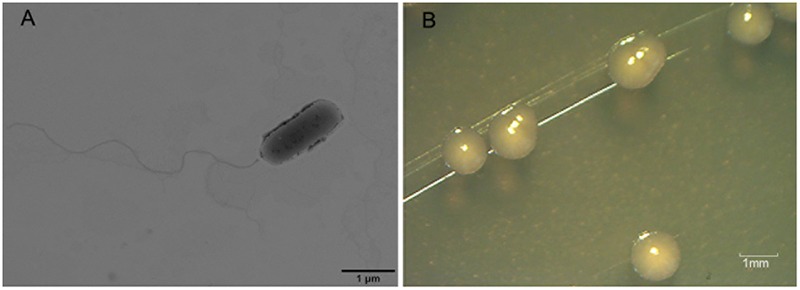
Transmission electron microscopy (TEM) image of endophytic bacterium L10 and its colonies on LB agar medium. **(A)** Transmission electron microscopy (TEM) image of rod-shaped endophytic isolate L10. **(B)** The circular and smooth colonies of endophytic bacterium L10 on LB agar medium.

**FIGURE 2:**
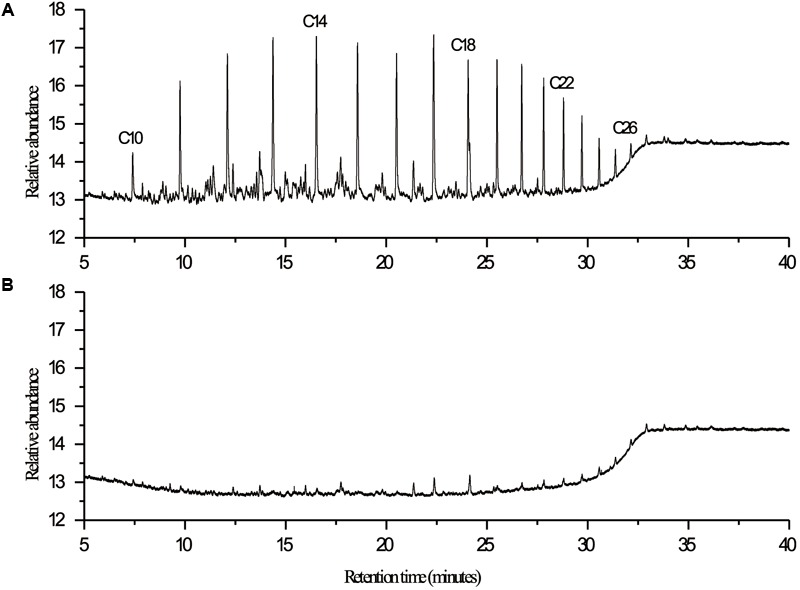
GC-FID chromatograms of diesel oil extracted at 7th day from **(A)** control and **(B)** experimental flask in biodegradation study with endophytic isolate L10. Degradation of diesel oil was carried out under aerobic conditions at 30°C in 300-mL Erlenmeyer flasks containing 100 mL of MS medium and 5 g/L of diesel oil. Control was performed by inoculating with the boiled cells of isolate. Hydrocarbons were extracted from the culture medium with an equal volume of *n*-hexane, analyzed using a gas chromatograph equipped with an FID detector (GC-FID, 7890A, Agilent, United States), and quantified in comparison to the *n*-alkane homologous series.

**FIGURE 3:**
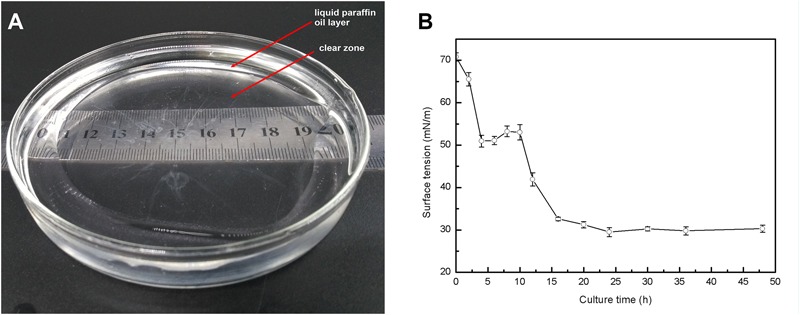
Production of biosurfactant by *P*. *aeruginosa* L10. **(A)** Oil displacement induced by the fermentation broth of a 48-h-old culture of strain L10 was measured using the oil spreading technique. **(B)** Surface tension of the fermentation broth as a function of time. Error bars indicate the standard deviations of three replicates.

Nineteen endophytic isolates were obtained from the roots, leaves, and stems of *P. australis*. Isolate L10, which we refer to as *P. aeruginosa* L10, was selected for this study because of its excellent hydrocarbon degradation, biosurfactant synthesis, and plant growth-stimulating attributes. *Pseudomonas aeruginosa* L10 is a Gram-negative, rod-shaped bacterium (**Figure 1A**) that forms circular and smooth colonies (**Figure 1B**) on LB agar medium. *Pseudomonas aeruginosa* L10 had a strong ability to degrade TPHs in diesel oil, with a degradation rate of 79.3% after 7 days of incubation in MS liquid medium supplemented with 5 g/L of diesel oil. By comparing the area peak of individual *n*-alkane from gas chromatograms (**Figure 2**), we also found that endophytic isolate L10 could degrade the C_10_–C_26_ *n*-alkanes of the diesel oil. In addition, *P. aeruginosa* L10 had a strong ability to degrade the common PAHs naphthalene, phenanthrene, and pyrene, with degradation rates of 79.7, 71.6, and 34.7%, respectively, after 10 days of incubation in MS medium with 200 mg/L of PAH. Moreover, an oil spreading assay and surface tension measurements of inocula indicated that *P. aeruginosa* L10 had the ability to produce biosurfactant. Diameters of the clearing zones on the oil surface obtained from the oil spreading assay with cell free supernatant of *P. aeruginosa* L10 were measured up to ∼80 mm (**Figure 3A**). However, no clearing zone with only medium was observed. *Pseudomonas aeruginosa* L10 could decrease the surface tension from 70.7 mN/m to 29.5 mN/m (**Figure 3B**) in the cell free supernatant.

We found that *P. aeruginosa* L10 has plant growth stimulating attributes, including siderophore and indole-3-acetic acid (IAA) production, along with 1-aminocyclopropane-1-carboxylic (ACC) deaminase activity. To quantify siderophore yield, we measured orange halo zone diameters in CAS medium 48 h post-inoculation. Siderophore zone diameters at 48 h were 4.1–4.5 cm. After a 72-h incubation period, the amount of IAA production by isolate L10 was 26.8 ± 7.4 mg/mL, as measured by the microplate method. *Pseudomonas aeruginosa* L10 could grow on DF minimal salt medium with ACC serving as the sole nitrogen source, indicating its ACC deaminase activity. The ACC deaminase enzyme activity of L10 was measured in bacterial extracts by quantifying the amount of alpha-KB produced during the deamination of ACC. The ACC deaminase activity of strain L10 was detected as 7496 ± 261 nmol alpha-KB/mg Pr⋅h.

**FIGURE 4:**
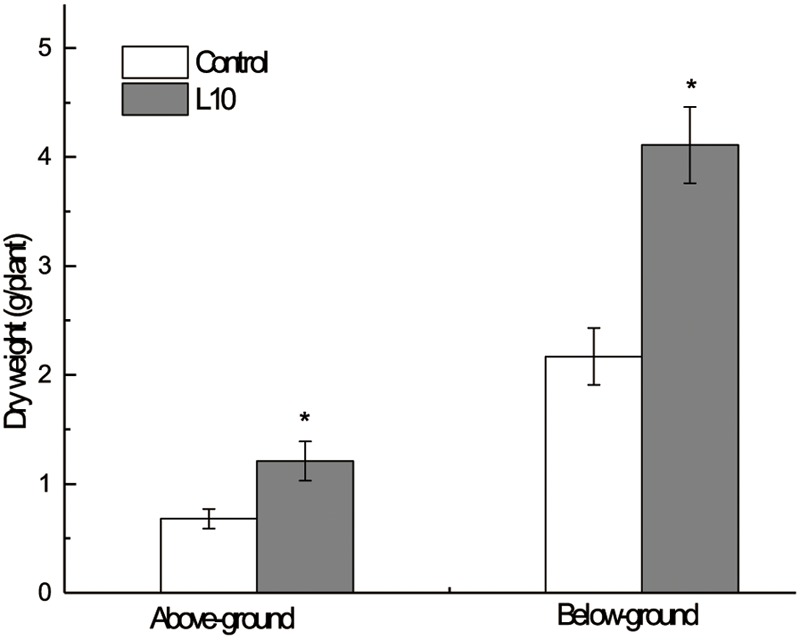
Effect of endophytic isolate L10 on the growth of the *Phragmites australis*. Bars indicate standard deviation (*n* = 6). Dried mass of inoculated plants was significantly greater (Student’s *t*-test; *P* < 0.05).

Moreover, pot experiments were carried out to verify the effect of *P. aeruginosa* L10 on plant growth promotion. After 120 days of growth, the reed plants were harvested and the biomass above and below the ground (dry weight) was measured. We found that aerial and underground biomass of inoculated plants was significantly higher than that of non-inoculated plants (*P* < 0.01); aerial biomass was elevated by 77.9%, and underground biomass was elevated by 89.4% (**Figure 4**), indicating that *P. aeruginosa* L10 can significantly promote reed growth both above and below the ground. Moreover, the soil TPH degradation rate in phytoremediation incubated with *P. aeruginosa* L10 was 58.9%, elevated by 21.7% over phytoremediation without incubation (37.2%).

### Complete Genome Sequencing of Endophytic *P. aeruginosa* L10

**FIGURE 5:**
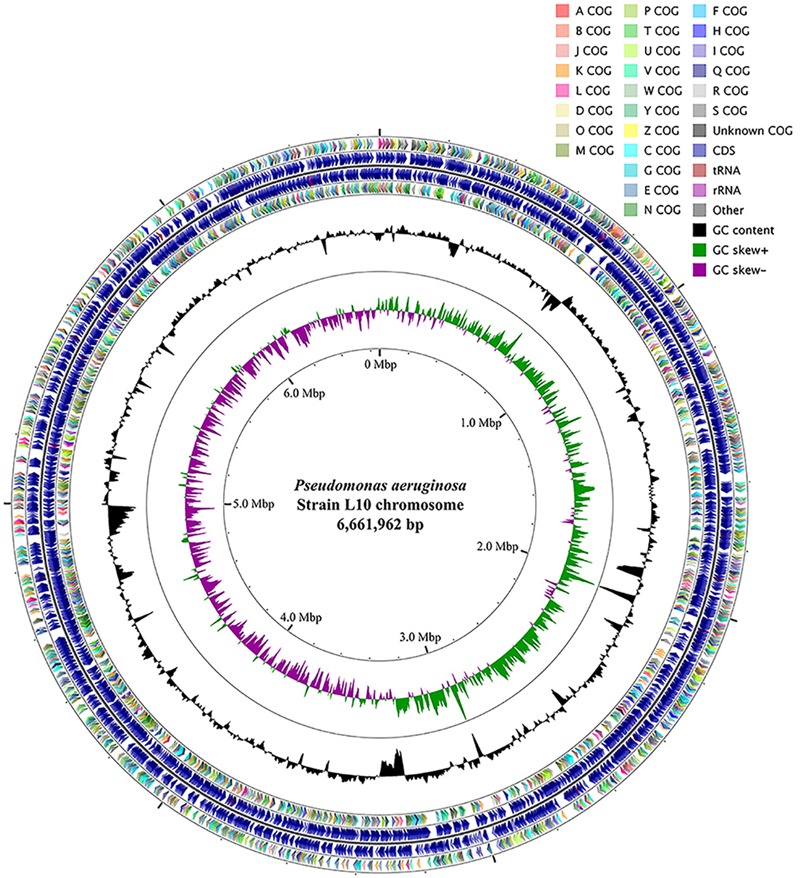
Circular genome map of *P. aeruginosa* L10. From outside to center, ring 1 and 4 show protein-coding genes oriented in the forward (colored by COG categories) and reverse (colored by COG categories) directions, respectively. ring 2 and 3 denote genes on forward/reverse strand; ring 5 shows G + C% content plot, and the inner-most ring shows GC skew, purple indicating negative values and olive, positive values. The circular map was generated with GCViewer (https://github.com/chewiebug/GCViewer, last accessed January 2017).

We sequenced the complete genome of isolate L10 and used this sequence to identify the isolate as *Pseudomonas aeruginosa*. The genome of *P. aeruginosa* L10 had one 6,661,962 bp circular chromosome (66.1% GC content; **Table 1**). NCBI’s Prokaryotic Genome Annotation Pipeline predicted 6,137 protein-coding genes, 12 rRNA genes (encoding four 23S rRNAs, four 16S rRNAs, and four 5S rRNAs), 62 tRNA genes, and 5 ncRNA genes, from a total of 6,307 genes (**Figure 5** and **Table 1**). The rRNA genes were arranged in four operons with the typical 16S-23S-5S rRNA pattern. Genes were affiliated to COG- and Pfam-based functions were 79.66 and 83.57%, respectively. Supplementary Table S1 shows the distribution of genes across their COG functional categories.

**Table 1:** General features of the genome of *P. aeruginosa* strain L10.

Attribute	Value	% of Total
Genome size (bp)	6,661,962	100.00
DNA coding (bp)	6,020,933	90.38
DNA G + C (bp)	4,405,868	66.13
DNA scaffolds	1	100.00
Total genes	6,307	100.00
Protein coding genes	6,137	97.30
RNA genes	79	1.25
Pseudo genes	91	1.44
Genes with function prediction	4912	77.88
Genes assigned to COGs	5024	79.66
Genes with Pfam domains	5,271	83.57
Genes with signal peptides	720	11.42
Genes with transmembrane helices	1,413	22.40
CRISPR repeats	2	0.03

### Functional Genomics of Endophytic *P. aeruginosa* L10

Genome annotation suggested the presence of genes related to hydrocarbon degradation, including genes encoding monooxygenase, dioxygenase, alcohol dehydrogenase, and aldehyde dehydrogenase. Genes related to the *n*-alkane degradation pathway were detected, such as genes encoding alkane 1-monooxygenase (*BWR11_13240, BWR11_19485*), 9 genes encoding aldehyde dehydrogenases, 5 genes encoding acyl-CoA synthetases, genes coding for rubredoxin reductase (*BWR11_30355*) and rubredoxin-NAD^+^ reductase (*BWR11_30 < 360*), genes coding for Rubredoxin-1 (*BWR11_30370*) and Rubredoxin-2 (*BWR11_30365*), 7 genes encoding methyl-accepting chemotaxis protein, and 7 genes coding for alcohol dehydrogenases (ADH). We also used AromaDeg^1^ (last accessed in May 2017) to detect genes putatively associated with the degradation of aromatic compounds. Five candidates were indicated: (1) three belonging to the benzoate family; (2) one belonging to the homoprotocatechuate family in the LigB superfamily; and (3) one belonging to the gentisate family.

A series of genes or gene clusters that may be involved in important biological functions were detected in the genome of endophytic *P. aeruginosa* L10. The genome annotation identified a potential rhamnolipid biosynthesis gene cluster, *rhlABRI* (*BWR11_07880* to *BWR11_07895*). Genome annotation also indicated the presence of an ACC deaminase gene (*BWR11_10155*). In addition, we also found seven tryptophan biosynthetic genes clustered at various chromosomal locations, including *trpAB* (*BWR11_00180* and *BWR11_00185*), *trpDC* (*BWR11_03465* and *BWR11_03470*), *trpE* (two copies: *BWR11_22230* and *BWR11_03280*), *trpF* (*BWR11_10025*), and *trpG* (two copies: *BWR11_03460* and *BWR11_22225*). Moreover, we detected two clusters of genes involved in the biosynthesis of siderophore, *pvcABCD* (pyoverdine siderophore; *BWR11_15175* to *BWR11_15190*) and *pchABCDREFG* (pyochelin siderophore; *BWR11_03850* to *BWR11_03885*).

### Phylogenetic Analysis of *P. aeruginosa* L10 and Other *P. aeruginosa* Strains

**FIGURE 6:**
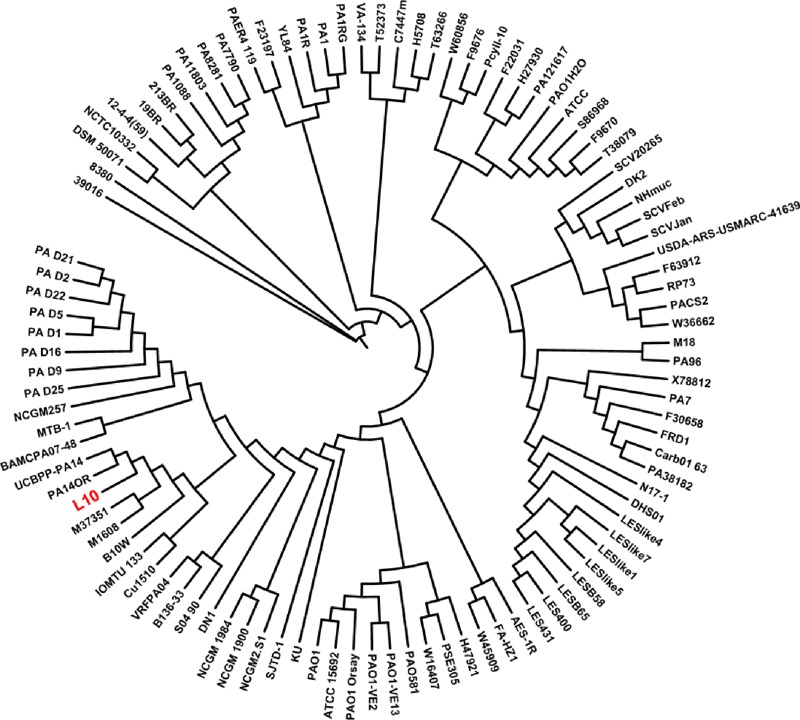
Phylogeny based on core SNPs of 101 strains of *P. aeruginosa*. We constructed the phylogenetic tree using kSNP3.0, and visualized it using iTOL. *P. aeruginosa* L10 is highlighted in red.

We used kSNP3.0 and the complete genomes of 101 *P. aeruginosa* strains (the genome of strain L10 and 100 publicly available genomes) to construct a phylogenetic tree; we visualized the tree using the iTOL software. Phylogenetic analysis of the 101 *P. aeruginosa* strains grouped strain L10 in a clade with seven other *P. aeruginosa* strains (B10W, Cu1510, IOMTU_133, M1608, M37351, UCBPP-PA14, and PA14OR; **Figure 6**). Alignment of the genomes of strain L10 and the reference strain PA01 (GenBank Accession No. NC_002516.2) using MUMmer 3.23 indicated relatively high sequence similarity (coverage 90%, similarity 99%; Supplementary Figure S1).

## Discussion

Phytoremediation with endophytic microbes is an important new means of improving bioremediation efficacy (Andreolli et al., 2013; Zhang et al., 2014). Isolation and selection of hydrocarbon-degrading and plant-growth-promoting endophytic bacteria from plants growing in contaminated sites could be particularly important for exploiting the remediation potential of plant-endophyte systems. The present study isolated an endophytic *P. aeruginosa* L10 from reed roots, and found that the isolate degraded hydrocarbons, synthesized biosurfactant, and stimulated plant growth via IAA synthesis, siderophore production, and ACC deaminase activity. *Pseudomonas* sp. are ubiquitous in soil, water, plants, animals, and humans, and have been studied for their versatile metabolic capacity to induce systemic resistance, promote plant growth, and suppress microbial pathogens in plants (Ma et al., 2017). Until recently, over 20 endophytic *Pseudomonas* sp. strains (Supplementary Table S2) with some of these functions beneficial to their plant host were isolated from various herbaceous, vine, and woody plants. To the best of our knowledge, strain L10 is the first endophytic *P. aeruginosa* isolated from the halophytic reed of the Yellow River Delta with the characteristics of hydrocarbon degradation, biosurfactant production, and plant growth promotion.

Alkanes and PAHs are toxic compounds ubiquitous in petroleum (Bakker et al., 2000). Despite their poor water solubility, they can be absorbed and accumulated by plants (Palmroth et al., 2002; Meudec et al., 2006). This study demonstrates that endophytic *P. aeruginosa* L10 can effectively degrade *n*-alkanes and PAHs (naphthalene, phenanthrene, and pyrene). Therefore, in this case, endophytic *P. aeruginosa* L10 may directly detoxify hydrocarbons in plants. Some studies demonstrated that endophytes can improve biodegradation by reducing the amount of contaminants in the growth substrate, as well as through reduction of evapotranspiration of contaminants (Barac et al., 2004; Germaine et al., 2009). Conversely, halobiotic reed is one of the most dominant plant species in the saline area of the Yellow River Delta, China (Wang et al., 2012). This plant species has been chronically exposed to petroleum hydrocarbons in the Yellow River Delta since Shengli Oilfield (the second largest oil-producing base of China and lies in the Yellow River Delta) exploitation began in 1964 (Nie et al., 2009) and might have adapted itself to the petroleum-polluted saline soils. Early reports indicated that reed had high potential for phytoremediation of petroleum pollution in Yellow River Delta (Wang et al., 2011). This study demonstrates *P. aeruginosa* L10 could increase the biomass of its host reed; the association between halophytic reed and *P. aeruginosa* L10 exerted a significant effect on TPH removal from crude oil-contaminated soils, compared to uninoculated plants. These results suggest an immense bioremediation potential for *P. aeruginosa* L10.

An important limiting factor on hydrocarbon biodegradation is the low bioavailability of these compounds. Hydrocarbon-degrading microbes might produce biosurfactant to overcome this problem (Kukla et al., 2014). In the present study, evidence from both an oil spreading assay and surface tension measurements of inocula indicated that endophytic *P. aeruginosa* L10 has the ability to produce biosurfactant. Kukla et al. (2014) also observed a number of *Pseudomonas* sp. in the endophytic community of *Lolium perenne*. Biosurfactants released by bacteria can significantly increase solubility of hydrocarbons, it is likely that plant uptake of hydrocarbons at the phytoremediation contaminated soil was relatively high. High uptake of hydrocarbons by plants would result in high selective pressure on endophytic bacterial communities, and degrader communities would become well established (Phillips et al., 2008).

The endophyte *P. aeruginosa* L10 produces siderophore, IAA, and ACC deaminase. Microbes with these features may promote plant growth (Rodriguez et al., 2008). Siderophore and IAA were found capable of stimulating plant biomass (Zhang et al., 2011), and the ACC deaminase has been proposed to play a key role in microbe–plant association (Hontzeas et al., 2004).

We sequenced the complete genome of endophytic *P. aeruginosa* L10, using high-throughput sequencing, to better understand the genetic bases of its functions (hydrocarbon degradation, biosurfactant synthesis, and plant growth promotion). A deeper understanding of the interaction between endophytic *P. aeruginosa* L10 and its plant host (*Phragmites australis*) could be achieved through the functional genomic analysis of the endophyte; this understanding will benefit the application of endophytic bacteria to phytoremediation.

Endophytic *P. aeruginosa* L10 was an efficacious hydrocarbon-degrading bacterium, which was detected degrade the C_10_–C_26_ *n*-alkanes of the diesel oil effectively, and degradation rate of TPHs was measured up to 79.3% after 7 days of incubation. Complete genomic sequence of *P. aeruginosa* L10 was detected to be abundant in genes involved in *n*-alkane degradation, including genes encoding alkane 1-monooxygenase, rubredoxin, rubredoxin reductase, alcohol dehydrogenase, aldehyde dehydrogenase, and acyl-CoA synthetase. The medium- and long-chain *n*-alkanes could be oxidized by alkane 1-monooxygenase (alkane hydroxylase) with the help of two soluble electron transfer proteins (a dinuclear iron rubredoxin and a mononuclear iron rubredoxin reductase). The terminal oxidation of alkanes by alkane hydroxylases generated primary fatty alcohols (Smits et al., 2003), which are further oxidized to aldehydes by an alcohol dehydrogenases (ADH) (Rojo, 2009). The aldehydes then were converted to carboxylic acids by aldehyde dehydrogenases (Van Beilen and Funhoff, 2007). The carboxylic acids then served as substrates for acyl-CoA synthetase, and the resulting acyl-CoA entered the β-oxidation pathway (Wentzel et al., 2007). We found a substantial number of genes encoding putative enzymes characteristic of β-oxidation, including acyl-CoA dehydrogenase (12 genes) and enoyl-CoA hydratase (14 genes). Moreover, we also detected genes putatively associated with the degradation of aromatic compounds in the complete genome of endophytic *P. aeruginosa* L10. The aromatic compounds, particularly PAHs (e.g., naphthalene, phenanthrene, and pyrene), are more toxic than aliphatic compounds to organismal and ecological security (Harayama et al., 1999). Our PAH biodegradation assay showed that *P. aeruginosa* L10 had a strong ability to degrade naphthalene, phenanthrene, and pyrene. The effective degradation of aromatic compounds of endophytic *P. aeruginosa* L10 indicated its broad application prospect in practice of phytoremediation.

Genome annotation suggested a potential rhamnolipid biosynthesis gene cluster in the genome, *rhlABRI* (*BWR11_07880* to *BWR11_07895*), which would be responsible for the biosurfactant synthesis. In the rhamnolipid pathway, the *rhlAB* genes (*BWR11_07880* and *BWR11_07885*; functioning as one operon) encode rhamnosyltransferase 1, while *rhlR* (*BWR11_07890*) and *rhlI* (*BWR11_07895*) are arranged in sequence and act as regulators of *rhlAB* expression (Lang and Wullbrandt, 1999). Rhamnolipid biosurfactants are surface-active compounds, produced by *P. aeruginosa*, that enhance the emulsification of hydrocarbons, increasing their solubility and their availability for microbial degradation (Rahman et al., 2002). We suggest that, in *P. aeruginosa* L10, the rhamnolipid *rhlABRI* gene cluster produces biosurfactants that promote the degradation of hydrocarbons.

We find that the endophyte *P. aeruginosa* L10 has plant growth-stimulating attributes, including siderophore and IAA release, along with ACC deaminase activity. Genome annotation identified two clusters of genes involved in the biosynthesis of siderophore: *pvcABCD* (*BWR11_15175* to *BWR11_15190*), and *pchABCDREFG* (*BWR11_03850* to *BWR11_03885*). The *pvcABCD* operon of *P. aeruginosa* encoded four proteins (PA2254, PA2255, PA2256, and PA2257) which were identified as being responsible for a step in the maturation of the chromophore of the peptide siderophore pyoverdine (Drake and Gulick, 2008). Expression of the *pchABCDREFG* operon was required for the synthesis of the pyochelin siderophore (Lee et al., 2013). In addition, genome annotation indicated seven tryptophan biosynthetic genes clustered at various chromosomal locations, including *trpAB* (*BWR11_00180* and *BWR11_00185*), *trpDC* (*BWR11_03465* and *BWR11_03470*), *trpE* (two copies: *BWR11_22230* and *BWR11_03280*), *trpF* (*BWR11_10025*), and *trpG* (two copies: *BWR11_03460* and *BWR11_22225*). Tryptophan biosynthetic genes are involved in IAA synthesis (Ge et al., 2006), which should be the mechanism of IAA biosynthesis by the endophytic *P. aeruginosa* L10. Genome annotation also indicated an ACC deaminase gene (*BWR11_10155*) in the *P. aeruginosa* L10 genome. ACC deaminase activity in plant-associated bacteria significantly enhanced nutrient uptake (Safronova et al., 2006) and increased plant biomass (Belimov et al., 2009), consistent with our finding that *P. aeruginosa* L10 increased plant growth.

## Conclusion

The endophyte *P. aeruginosa* L10 degrades petroleum hydrocarbons, produces biosurfactants, and promotes plant growth of *Phragmites australis*. This study used high-throughput sequencing and functional genomics to elucidate the mechanisms of hydrocarbon degradation, biosurfactant synthesis, and plant growth promotion by endophytic *P. aeruginosa* L10. The complete genome we have here presented will serve as a genetic basis for further work on this endophytic bacterial strain and will thus be of considerable value for further studies of the industrial and environmental applications of *P. aeruginosa* L10.

## Author Contributions

TW designed the experiments. TW, JX, WX, and HY performed the experiments. TW and XL analyzed the data and wrote the manuscript. ZY and CS provided technical assistance to TW. All the authors read and approved the final manuscript.

## Conflict of Interest Statement

The authors declare that the research was conducted in the absence of any commercial or financial relationships that could be construed as a potential conflict of interest.

## Footnotes

1 http://aromadeg.siona.helmholtz-hzi.de
