# Supplementary material for: Pseudomonas aeruginosa L10: A Hydrocarbon-Degrading, Biosurfactant-Producing, and Plant-Growth-Promoting Endophytic Bacterium Isolated From a Reed (Phragmites australis)
Source: Front Microbiol. 2018 May 25;9:1087. doi: 10.3389/fmicb.2018.01087 (PMC5980988; doi:10.3389/fmicb.2018.01087)
Supplement: Supplementary file 1 [file Table_1.DOCX]

Supplementary Material

*Pseudomonas aeruginosa* L10: A Hydrocarbon-degrading, Biosurfactant-producing, and Plant-growth-promoting Endophytic Bacterium Isolated from a Reed (*Phragmites australis*)

**Tao Wu, Jie Xu, Wenjun Xie, Zhigang Yao, Hongjun Yang, Chunlong Sun, Xiaobin Li Correspondence:** Tao Wu e-mail: [wtsdbz@hotmail.com](mailto:wtsdbz@hotmail.com); Wenjun Xie xwjeric@163.com

## 1. SupplementaryTable

**Supplementary Table 1 |** Number of genes associated with general COG functional categories

| Code | Value | %age | Description |
| --- | --- | --- | --- |
| J | 277 | 4.51 | Translation, ribosomal structure and biogenesis |
| A | 2 | 0.03 | RNA processing and modification |
| K | 551 | 8.98 | Transcription |
| L | 144 | 2.35 | Replication, recombination and repair |
| B | 3 | 0.05 | Chromatin structure and dynamics |
| D | 57 | 0.93 | Cell cycle control, cell division, chromosome partitioning |
| V | 139 | 2.26 | Defense mechanisms |
| T | 409 | 6.66 | Signal transduction mechanisms |
| M | 310 | 5.05 | Cell wall/membrane/envelope biogenesis |
| N | 158 | 2.57 | Cell motility |
| U | 183 | 2.98 | Intracellular trafficking, secretion, and vesicular transport |
| O | 231 | 3.76 | Posttranslational modification, protein turnover, chaperones |
| C | 342 | 5.57 | Energy production and conversion |
| G | 269 | 4.38 | Carbohydrate transport and metabolism |
| E | 542 | 8.83 | Amino acid transport and metabolism |
| F | 120 | 1.96 | Nucleotide transport and metabolism |
| H | 267 | 4.35 | Coenzyme transport and metabolism |
| I | 317 | 5.17 | Lipid transport and metabolism |
| P | 351 | 5.72 | Inorganic ion transport and metabolism |
| Q | 207 | 3.37 | Secondary metabolites biosynthesis, transport and catabolism |
| R | 623 | 10.15 | General function prediction only |
| X | 101 | 1.65 | Mobilome: prophages, transposons |
| Z | 1 | 0.02 | Cytoskeleton |
| W | 57 | 0.93 | Extracellular structures |
| S | 367 | 5.98 | Function unknown |
| - | 1113 | 18.14 | Not in COGs |
