# Supplementary material for: Pseudomonas aeruginosa L10: A Hydrocarbon-Degrading, Biosurfactant-Producing, and Plant-Growth-Promoting Endophytic Bacterium Isolated From a Reed (Phragmites australis)
Source: Front Microbiol. 2018 May 25;9:1087. doi: 10.3389/fmicb.2018.01087 (PMC5980988; doi:10.3389/fmicb.2018.01087)
Supplement: Supplementary file 2 [file Table_2.docx]

Supplementary Material

*Pseudomonas aeruginosa* L10: A Hydrocarbon-degrading, Biosurfactant-producing, and Plant-growth-promoting Endophytic Bacterium Isolated from a Reed (*Phragmites australis*)

**Tao Wu, Jie Xu, Wenjun Xie, Zhigang Yao, Hongjun Yang, Chunlong Sun, Xiaobin Li Correspondence:** Tao Wu e-mail: [wtsdbz@hotmail.com](mailto:wtsdbz@hotmail.com); Wenjun Xie xwjeric@163.com

## 1. SupplementaryTable

**Supplementary Table 2 |** Summary of endophytic Pseudomonas sp. strains

| Endophytic strain | Plant host | Functions | GenBank | Reference |
| --- | --- | --- | --- | --- |
| *P. aeruginosa* L10 | Phragmites australis | Hydrocarbon degradation, Biosurfactant synthesis, Plant growth promotion | CP019338 | This study |
| *P. aeruginosa* AL2-14B | *Achyranthes aspera* L. | Antioxidant activity, Plant growth promotion | KY0879823.1 | Devi *et al.,* 2017 |
| *P. aeruginosa* BP35 | *Piper nigrum* | Biosurfactant synthesis, Multiple antibiotics resistance | JN159935 | Kumar *et al.,* 2013 |
| *P. aeruginosa* PM389 | *Pennisetum glaucum* (pearl millet) | Plant growth promotion | JF899310 | Gupta *et al.*, 2013 |
| *P. aeruginosa* BRRI54 | *Brachiaria mutica* | Hydrocarbon degradation, Plant growth promotion | KJ620860 | Fatima *et al.*, 2015 |
| *P. aeruginosa* PW09 | *Triticum aestivum* | Plant growth promotion, Antagonistic activitiy | - | Pandey *et al.*, 2012 |
| *P. aeruginosa GSE 18* | *Arachis hypogaea* | Antibacterial activity | - | Krishna Kishore *et al.*, 2005 |
| *P. aeruginosa* GSE 19 | *Arachis hypogaea* | Antibacterial activity | - | Krishna Kishore *et al.*, 2005 |
| *P. putida* SV699 | *S. alterniflora* | Hydrocarbon degradation | KP757587 | Zheng *et al.*, 2018 |
| *P. aeruginosa* (UPMP3) | Oil palm | Antibacterial activity | - | Siddiqui *et al.*, 2009 |
| *P. aeruginosa* RRA | *Oryza sativa* L. | Chlorpyrifos degradation, Plant growth promotion | - | Feng *et al.*, 2017 |
| *P. aeruginosa* UICC B-40 | *Neesia altissima* | Antibacterial activity | LC159298.1 | Pratiwi *et al.,* 2017 |
| *Pseudomonas* sp.J4AJ | *Scirpus triqueter* | Hydrocarbon degradation | KF668329 | Zhang *et al.,* 2014 |
| *Pseudomonas* sp. Ph6 | *Trifolium pratense* L. | Hydrocarbon degradation | KF741207 | Sun *et al.,* 2014 |
| *P. fluorescen* YsS6 | Tomato | Plant growth promotion | N118641 | Ali *et al.,* 2014 |
| *P. migulae* 8R6 | Tomato | Plant growth promotion | JN118619 | Ali *et al.,* 2014 |
| *P. viridiflava* CDRTc14 | *Lepidium draba* L. | Bioherbicidal activity, Plant growth promotion | MBPF00000000 | Samad *et al.,* 2017 |
| *Pseudomonas* sp. ITRI53 | Italian ryegrass | Hydrocarbon degradation | - | Afzal *et al.,*  2011 |
| *Pseudomonas* sp. HU002 | Willow | Plant growth promotion, increased Cd resistance | - | Weyens *et al.*, 2013 |
| *Pseudomonas* sp. P21 | Tomato | Antibacterial activity | NZ_LWBV00000000.1 | Ma *et al.,* 2017 |
