# Supplementary material for: Pseudomonas aeruginosa L10: A Hydrocarbon-Degrading, Biosurfactant-Producing, and Plant-Growth-Promoting Endophytic Bacterium Isolated From a Reed (Phragmites australis)
Source: Front Microbiol. 2018 May 25;9:1087. doi: 10.3389/fmicb.2018.01087 (PMC5980988; doi:10.3389/fmicb.2018.01087)
Supplement: Supplementary file 3 [file Image_1.PDF]

Supplementary Material

Pseudomonas aeruginosa L10: A Hydrocarbon-degrading, Biosurfactant-producing, and Plant-growth-promoting Endophytic Bacterium Isolated from a Reed (*Phragmites australis*)

Tao Wu, Jie Xu, Wenjun Xie, Zhigang Yao, Hongjun Yang, Chunlong Sun, Xiaobin Li
Correspondence: Tao Wu e-mail: wtsdbz@hotmail.com; Wenjun Xie xwjeric@163.com

1. Supplementary Figures

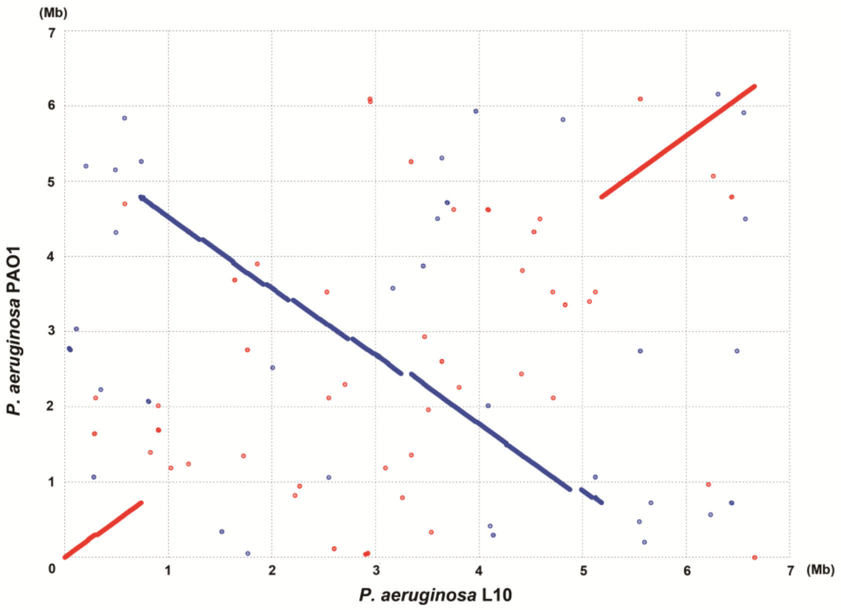

Supplementary Figure 1 | Dot plot comparison of the genomes of *P. aeruginosa* L10 and reference strain PAO1. Nucleotide-based alignments were performed with MUMmer 3.23 (Kurtz et al., 2004). Dot plots were generated with mummerplot and gnuplot.
